# The Relationship between Academic Achievement and the Emotional Well-Being of Elementary School Children in China: The Moderating Role of Parent-School Communication

**DOI:** 10.3389/fpsyg.2016.00948

**Published:** 2016-06-24

**Authors:** Bo Lv, Huan Zhou, Xiaolin Guo, Chunhui Liu, Zhaomin Liu, Liang Luo

**Affiliations:** ^1^Collaborative Innovation Center of Assessment toward Basic Education Quality, Beijing Normal University, BeijingChina; ^2^School of Sociology, China University of Political Science and Law, BeijingChina

**Keywords:** academic achievement, emotional well-being, parental involvement, parent–school communication, elementary school children

## Abstract

The relationship between academic achievement and the subjective well-being of elementary school children has received increasing attention. However, previous research on the relationship between these variables has yielded inconsistent conclusions – possibly due to the presence of potential moderating variables. This study investigated the relationship between the academic achievement and the emotional well-being (positive and negative affect) of elementary school children in China and the moderating effect of parent–school communication on this relationship. A total of 419 elementary school students and their parents participated. The elementary students’ positive and negative affect, their academic achievement on both midterm and final examinations of the most recent semester, and the frequency of parent–school communication were assessed. Academic achievement of elementary students was positively correlated with positive affect and negatively correlated with negative affect. Parent–school communication significantly moderated this relationship. Regardless of positive or negative affect, the correlation was only significant in the high parent–school communication group (one standard deviation higher than the mean) and in the mean group, whereas in the low parent–school communication group, no association was observed. These results indicate that parental engagement with school impacts both the academic achievements and subjective well-being of children in China.

## Introduction

With the continuous dissemination of research findings from positive psychology, subjective well-being is receiving increasing attention. Subjective well-being, which primarily refers to the perceptions, evaluations, and desires that individuals have regarding their lives (Campbell et al., 1976), consists of two basic components: life satisfaction and emotional experience. The former involves the cognitive assessment of total quality of life, i.e., an individual’s overall satisfaction level with their personal life. The latter involves the emotional experience of personal life, including positive and negative affect. Some researchers collectively refer to positive and negative affect as emotional well-being (Schimmack et al., 2008; Kahneman and Deaton, 2010). A series of studies has shown many benefits for individuals that enjoy high subjective well-being, including health, life (Diener and Chan, 2011), work, income, and friendship (Diener and Ryan, 2009).

Previous studies of subjective well-being have primarily focused on adults; however, increasingly researchers are focusing on children and adolescents. Studies have shown that higher subjective well-being in childhood is positively correlated with physical and mental health, excellent interpersonal relationships, and even future career success (Park, 2004). By contrast, lower well-being typically indicates a higher likelihood of risk behaviors, such as drug and alcohol abuse and violent behaviors (Proctor et al., 2009). Because school-age children and adolescents spend most of their time in school, their subjective well-being is largely influenced by a variety of academic factors (Suldo and Huebner, 2006; Stiglbauer et al., 2013). In today’s highly competitive schools, students face increasing pressure to achieve academic success (Crede et al., 2015). Therefore, the relationship between academic achievements and the subjective well-being of children and adolescents has received great attention from researchers (Quinn and Duckworth, 2007; Suldo et al., 2008).

### The Relationship between Academic Achievement and the Subjective Well-Being of Children and Adolescents

Academic achievement can influence the future education and career choices of children and adolescents (Rana and Mahmood, 2010; Flashman, 2012). Poor academic performance causes children and adolescents to feel pessimistic and disappointed about their future and leads teachers and parents to exert pressure, which negatively influences the subjective well-being of children and adolescents. Moreover, many studies have indicated a correlation between academic achievement and the subjective well-being of children and adolescents. For example, Kirkcaldy et al. (2004) analyzed the Program for International Student Assessment (PISA) to investigate the relationship between academic achievement and the subjective well-being of high school students in 30 countries (using life satisfaction and negative affect as indicators of subjective well-being). Academic achievement was positively correlated with life satisfaction and negatively correlated with negative affect at the country level. Studies of high school students in Germany and the USA have shown that academic achievement is positively correlated with life satisfaction (Suldo et al., 2008; Crede et al., 2015).

Although many studies have found a relationship between academic achievement and the subjective well-being of children and adolescents, other studies have found inconsistent results. For example, Huebner (1991) investigated students in grades 5–7and found no significant correlation between academic achievement and life satisfaction. Huebner and Alderman (1993) performed a comparative analysis between elementary school students with learning difficulties and students with normal learning and observed significant differences in life satisfaction. Moreover, the latest longitudinal study found that academic achievement was not correlated with adolescents’ moods but that academic achievement can predict life satisfaction 1 year later (Steinmayr et al., 2015). Although these inconsistent results may be due in part to instrumentation or sample differences, the relationship between academic achievement and subjective well-being may also differ based on certain moderating variables.

### The Moderating Effect of School-Based Parental Involvement on the Relationship between Academic Achievement and the Subjective Well-Being of Children and Adolescents

Family provides sustained and stable resources for the lifelong development of children and adolescents and is a major factor affecting their outcomes (Berkowitz et al., 2015; Hill, 2015). Many studies have indicated that parental factors play an important role in the academic achievement (Cheung and Pomerantz, 2012; Stright and Yeo, 2014), life satisfaction (Leung et al., 2004), and emotional function (Cheung and Pomerantz, 2011; Wang et al., 2014) of children. However, only one study has considered the moderating function of the family on the relationship between academic achievement and subjective well-being. Crede et al. (2015) compared the high school education level attained by students and their parents and found that mothers’ education level moderated the relationship between the academic achievement and life satisfaction of adolescents. The academic achievement of adolescents with mothers who attained high education levels predicted life satisfaction, whereas no predictive effect was observed for adolescents whose mothers attained low education levels; however, parental education levels of parents do not directly influence adolescents. Crede et al. (2015) also expressed this perspective and concluded that researchers in this field should investigate the role of parental involvement in education.

School-based parental involvement is a factor of parental involvement in education that has received more attention. Students’ academic achievement is enhanced by parents through parent–school interaction, which generally includes parent–school communication, involvement in volunteer activities in schools, and participation in school management (Hill and Tyson, 2009). School-based parental involvement can transmit the importance of education to children and can help children to understand the importance of academic achievements (Lareau, 2000). Due to the influences of Confucianism in China, learning is not only in terms of acquiring knowledge and skills but also inconnection with morality (Li, 2002, 2005). Learning is also a process that involves acquiring qualities such as diligence, persistence, and concentration (Cheung and Pomerantz, 2011). Only children with excellent academic achievement will be labeled “good children” in China. Therefore, academic achievement has important social meaning for Chinese children. In addition, during the interaction process between parents and teachers, forming a consensus on academic and behavioral norms is easy. When the information acquired by children from the family and the school is consistent, this information will be become clearer and more precise, which enables children to more clearly understand the academic expectations of parents and teachers (Hill and Taylor, 2004). Therefore, frequent parent–teacher communications will help children internalize both parents’ and teachers’ academic expectations, thus strengthening the relationship between academic achievement and well-being in China. Furthermore, through school-based parental involvement in education, parents can acquire extensive information about children in school, providing a better understanding of children’s academic environment, which will cause parents to adjust behaviors based on academic achievement (Hill and Taylor, 2004). When children demonstrate poor academic achievement, Chinese parents may enforce more rigorous discipline and adopt more controlling parenting behaviors, reducing such children’s subjective well-being. Conversely, for children with higher academic achievement, Chinese parents may be warmer and express more support, resulting in higher subjective well-being for such children. The above reasons may lead school-based parental involvement to have a moderating effect on the relationship between academic achievement and the subjective well-being of children and adolescents in China. Many studies have shown that school-based parental involvement influences the academic achievement of students (for a review, see Hill and Tyson, 2009). In addition, compared with family based involvement, school-based involvement is more closely associated with the emotional development of children and adolescents. For example, the longitudinal study of Wang and Sheikh-Khalil (2014) demonstrated that school-based involvement can not only effectively reduce depression symptoms in adolescents but also increase their emotional engagement levels, thus enhancing academic achievements. These previous studies indirectly suggest that parental school-based involvement may exert a moderating effect on the relationship between academic achievement and subjective well-being. However, no study has yet directly addressed this issue to our knowledge.

School-based parental involvement has many forms; however, previous studies have shown that Chinese parents rarely participate in school volunteer activities and school management activities. A major form of school-based parental involvement is parent–school communication (Wu et al., 2013). Therefore, this study used parent–school communication as the major indicator of school-based involvement.

Previous research on the relationship between academic achievement and subjective well-being has primarily focused on adolescents (Suldo et al., 2008; Crede et al., 2015), and research on elementary school students is relatively scarce. In addition, previous studies have primarily focused on the cognitive component of well-being, i.e., life satisfaction, but have ignored emotional well-being. Previous studies have shown that although these two factors are associated, they are not perfectly correlated (Schimmack et al., 2008; Kahneman and Deaton, 2010). Moreover, some studies have suggested that emotional well-being is more variable than life satisfaction and more susceptible to the influence of school experience (Diener et al., 2003).

We investigated elementary school students in grades 4–6 in China to investigate the relationship between academic achievement and emotional well-being; furthermore, we controlling for gender and class level and explored whether parent–school communication moderates this relationship. It was hypothesized that (1) academic achievement of elementary school students are positively correlated with positive affect and negatively correlated with negative affect and that (2) parent–school communication may moderate the relationship between academic achievement and the affect of children, academic achievement and affect may be just correlated when parents have a higher frequency of parent–school communication.

## Materials and Methods

### Ethic Statement

All procedures in this study were approved by the Institutional Review Board of the Collaborative Innovation Center of Assessment toward Basic Education Quality, Beijing Normal University.

### Sample and Procedure

Two classes of students in grades 4, 5, and 6 in an elementary school in Liaocheng, Shandong, China, and one of their parents were randomly selected for investigation. Liaocheng is located in western Shandong Province. In 2013, the per capita annual disposable income of Liaocheng (18,085 Chinese Yuan/person) was equivalent to the national average (18,310.8 Chinese yuan/person). The educational level of elementary schools was equivalent to the national average level; for example, the pupil-teacher ratio of Liaocheng in 2013 was 17.96, and the national average was 16.76 (China Statistical Yearbook, 2014; Shandong province Statistical Yearbook, 2014). We distributed 510 surveys (each survey included a student and a parent component); 419 (82%) sets of parent–child surveys were returned (the analysis only included those returned surveys that included both the parent and student components). Among valid student questionnaires, there were 233 male students (55.61%) and 186 female students (44.39%), and the mean student age was 10.97 years. Among valid parent questionnaires, there were 157 fathers (34.47%) and 261 mothers (62.29%). One person did not report parent gender (0.24%). The mean parental age was 38.44 years.

Students completed the questionnaires in class. Parental questions were taken home by children on the previous day and returned the following day to the principle investigator. A cover letter inviting parents to participate and explaining the items was taken home by students along with the questionnaire.

### Measures

#### Academic Achievement

Academic achievement across three subjects (Chinese language, mathematics, and English language), determined by performance on the midterm and final examinations of the most recent semester was used. Original scores ranged from 0 to 100. Class level was used as a unit, and scores were transformed into standard achievement. Finally, standard achievement in the three subjects was averaged and used as the indicator of student academic achievement (Cheung and Pomerantz, 2011) (the averaged standard score ranged from -3.37 to 0.99). Standard scores between the three subjects were moderately correlated: Chinese language and English language, *r* = 0.58; Chinese language and mathematics, *r* = 0.60; and English language and mathematics, *r* = 0.58. Therefore, composite scores of academic achievement were calculated (Stright and Yeo, 2014).

#### Parent–School Communication

Parent–school communication was measured using seven questions (question numbers 2, 3, 9, 11, 21, 25, and 27 in the original questionnaire) from the parent–school communication dimension of the “Parental involvement in primary school children education questionnaire” (parent version) formulated by Wu et al. (2013), e.g., “I communicate with teachers regarding my child’s homework.” Four-point scoring was used (1 = never, 2 = seldom, 3 = sometimes, and 4 = usually). The Cronbach’s α of the seven questions was 0.87. Confirmatory factor analyses (CFAs) were conducted using structural equation modeling (SEM) in Amos 20.0. The model fit statistics for the parent–school communication questionnaire were all acceptable: χ^2^ = 40.44, *df* = 14, *p* < 0.001, χ^2^/*df* = 2.89, CFI = 0.98, TLI = 0.96, RMSEA = 0.07.

#### Positive and Negative Affect

The positive and negative affect scale compiled by Watson et al. (1988) was used to assess children’s positive and negative affect. The scale included nine descriptive terms for each positive and negative affect experience (such as happy and ashamed). Using these terms, participants were asked to report the affect they experienced over the previous week on a five-point scale (1 = very weak or no, 2 = very little, 3 = moderate, 4 = more, 5 = very strong). The Cronbach’s α of the positive and negative affect questions in present study were 0.80 and 0.82, respectively.

#### Demographic Variables

The gender and class level of children were obtained from the children’s questionnaires, and educational levels attained by parents were obtained from the parents’ questionnaires. Because the classification of occupational reputation in Chinese society is controversial (Ren, 2010), it was difficult to effectively rank the occupational reputation of parents. Therefore, we did not consider the influence of parent occupational reputation in the analyses. Family income was reported by parents on their questionnaire (missing *n* = 69). An analysis showed that family income did not significantly correlate with other variables (positive affect: *r* = 0.09, *p* = 0.09; negative affect: *r* = -0.06, *p* = 0.62; academic achievement: *r* = -0.01, *p* = 0.87; parent–school communication: *r* = 0.03, *p* = 0.55) and therefore was not included in the final regression.

### Data Analysis

Two variables, parent–school communication and educational level attained by parents, were obtained from the parent questionnaires; however, 91 students completed a survey but did not return parent questionnaires. To compare the students who did and did not return a parent survey, a *t*-test was performed on academic achievement, positive affect, and negative affect, and no significant differences were observed (*ps* > 0.05). There were no more than 3% missing data for any variable included in the analysis. Little’s MCAR test showed that the missing data of all variables were randomly distributed (*ps* > 0.05). Missing values were computed using full information maximum likelihood (FIML).

Hierarchical multiple regression analysis was performed to examine the moderating effect of parent–school communication on the relationship between academic achievement and children’s subjective well-being. Prior to regression, the academic achievement of children and parental parent–school communication were centralized (subtract the mean; Aiken and West, 1991).

## Results

**Table 1** presents the descriptive statistics and the correlations between variables. Academic achievement was positively correlated with positive affect (*r* = 0.18, *p* < 0.01) and negatively correlated with negative affect (*r* = -0.22, *p* < 0.01). Among the demographic variables, education level attained by parents was positively correlated with positive affect and negatively correlated with negative affect. Class level was negatively correlated with positive affect, and gender was negatively correlated with negative affect.

**Table 1:** Descriptive statistics and correlations between variables.

Variables	Gender	Class level	Mother education	Father education	School achievement	Parent-school communication	Positive affect	Negative affect
(1) Gender	—							
(2) Class level	0.43	—						
(3) Mother’s education	-0.02	-0.08	—					
(4) Father’s education	0.02	-0.09	0.67*	—				
(5) School achievement	0.26**	0	0.16*	0.16**	—			
(6) Parent–school communication	-0.03	-0.27*	0.14**	0.09	0.01	—		
(7) Positive affect	0.024	-0.20*	0.10*	0.11*	0.18**	0.12*	—	
(8) Negative affect	-0.12*	0.048	-0.18	-0.17**	-0.22**	-0.01	-0.26**	—
*M*	1.45	5.10	3.02	3.21	0.00	2.61	4.03	2.05
*SD*	0.50	0.83	1.17	1.18	0.77	0.61	0.73	0.74

*p < 0.05; **p < 0.01.

To validate whether parent–school communication moderated the relationship between academic achievement and children’s affect, positive and negative affect were separately used as dependent variables in two hierarchical multiple regressions. Children’s gender and class level were entered in the first step, education level attained by parents was entered in the second step, and parent–school communication and the academic achievement of children were entered in the third step to investigate whether academic achievement might predict children’s affect after controlling for related variables. Finally, the interaction between academic achievement and parent–school communication was entered. Because different correlation strengths were observed between many of the predictive variables, a multiple collinearity diagnosis was performed on the predictive variables during the regression analysis. Tolerance among all the predictive variables was higher than 0.5, and the variance inflation factors (VIFs) were all lower than 2, indicating that there were no severe multiple collinearity problems among the predictive variables (Fox, 1991).

**FIGURE 1:**
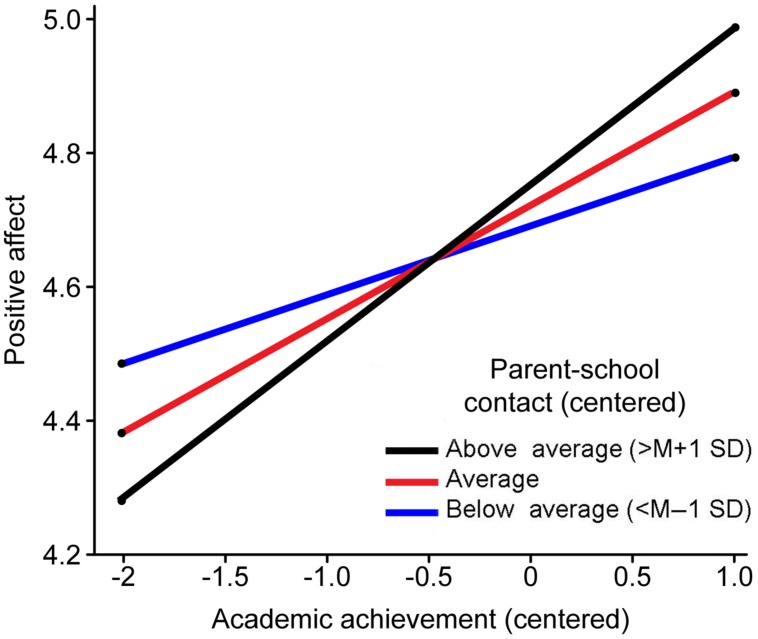
**Parent–school communication as a moderator of children’s academic achievement and positive affect**.

**Table 2** shows the hierarchical multiple regression results. For the positive affect regression, the first step showed that the main effect of class level was significant (β = -0.20, *p* < 0.01). As the class level increased, children’s positive affect decreased. In the second step, the education level attained by parents did not significantly predict the positive affect of children. In the third step, only academic achievement predicted the positive affect of children (β = 0.18, *p* < 0.01). The interaction between academic achievement and parent–school communication in the fourth step also reached significance. To understand the meaning of the interactive term, online resources provided by Preacher et al. (2006) were used in a further simple slope analysis. For parent–school communication, the mean values, the values one standard deviation above, and the values one standard deviation below were grouped. As expected, in the high scoring group (simple slope = 0.24, *t* = 3.64, *p* < 0.01) and the mean group (simple slope = 0.17, *t* = 3.80, *p* < 0.01), academic achievement positively predicted the positive affect of children. However, in the low scoring group, no significant predictive effect was observed (simple slope = 0.10, *t* = 1.57, *p* > 0.05; **Figure 1**).

**Table 2:** Hierarchical regression analyses predicting children’s positive and negative affect.

	Positive affect			Negative affect		
Variables	*B*	*SE B*	β	*B*	*SE B*	β
**Step 1**	*R*^2^ = 0.04**			*R*^2^ = 0.02*		
Gender	0.05	0.07	0.03	-0.17	0.07	-0.12*
Class level	-0.18	0.04	-0.20**	0.04	0.04	-0.05
**Step 2**	Δ*R*^2^ = 0.008			Δ*R*^2^ = 0.03*		
Mother’s education	0.03	0.04	0.04	-0.07	0.04	-0.11
Father’s education	0.03	0.04	0.06	-0.06	0.04	-0.09
**Step 3**	Δ*R*^2^ = 0.03**			Δ*R*^2^ = 0.03*		
School achievement	0.17	0.05	0.18**	-0.17	0.05	-0.18**
Parent–school communication	0.07	0.06	0.06	0.02	0.06	0.02
**Step 4**	Δ*R*^2^ = 0.01**			Δ*R*^2^ = 0.01*		
School achievement × Parent-school communication	0.18	0.08	0.11*	-0.20	0.08	-0.11*

*p < 0.05; **p < 0.01.

**FIGURE 2:**
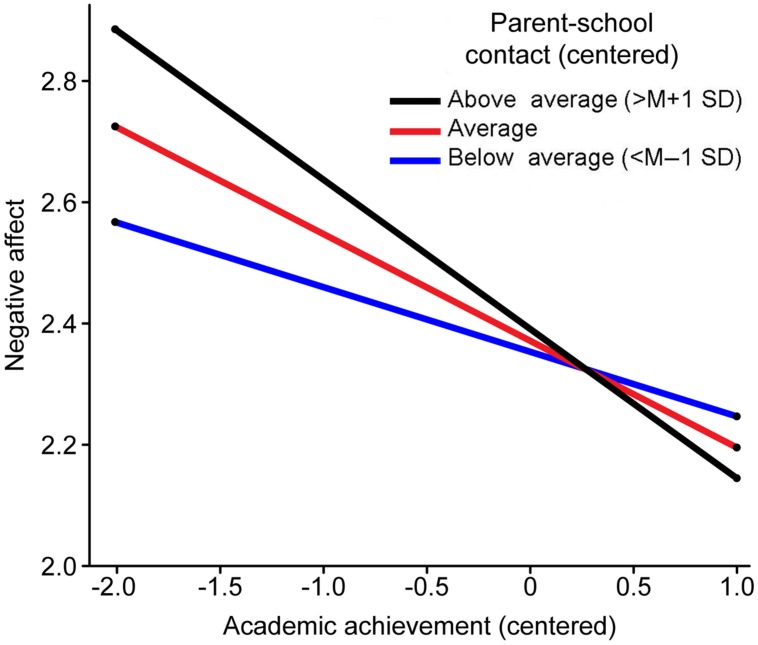
**Parent–school communication as a moderator of children’s academic achievement and negative affect**.

In the negative affect regression, the results from the first step showed that the main effect of gender was significant (β = -0.12, *p* < 0.05), indicating that compared with girls, boys had more negative affect. In the second step, educational level attained by parents was not significantly predictive of children’s negative affect. In the third step, only academic achievement negatively predicted negative affect. In the fourth step, the relation between academic achievement and parent–school communication reached significance, indicating that parent–school communication moderated the effect of academic achievement on negative affect. Simple slope analyses showed that academic achievement in the high parent–school communication group (simple slope = -0.24, *t* = -3.64, *p* < 0.01) and the mean group (simple slope = -0.17, *t* = -3.8, *p* < 0.01) negatively predicted the negative affect of children; however, it did not significantly predict negative affect in the low parent–school communication group (simple slope = -0.10, *t* = -1.57, *p* > 0.05; **Figure 2**).

## Discussion

This study explored the relationship between academic achievement and the emotional well-being (measured as positive and negative affect) of elementary school children and the moderating effect of parent–school communication in Chinese culture. After demographic variables were controlled for, academic achievement still predicted the emotional well-being of children. Parent–school communication significantly moderated the relationship between academic achievement and positive and negative affect.

### Relationship between Academic Achievement and Emotional Well-Being

Hierarchical multiple regression showed that class level negatively predicted the positive affect of children but did not predict negative affect. This conclusion is consistent with Kuppens et al. (2008), who showed that compared with negative affect, the relationship between positive affect and life satisfaction was much stronger. Previous studies have shown that children’s life satisfaction commonly decreases with age (Klocke et al., 2014); therefore, positive affect and life satisfaction exhibited similar trends with age. Gender negatively predicted children’s negative affect, suggesting that boys had more negative affect than girls. In previous studies, gender differences in the subjective well-being of children have been controversial. Some studies did not find gender differences (Seligson et al., 2003; Huebner et al., 2006), whereas others found that girls had higher subjective well-being (Bradshaw et al., 2011; Casas et al., 2013) or that boys had much higher subjective well-being (Rees et al., 2010). One possible reason for the results in our study is that compared with girls, boys at elementary school age commonly have higher activity levels and fewer compliance behaviors (Stright and Yeo, 2014); in addition, combined with the correlation between academic achievement and gender (*r* = 0.26), academic achievement in boys at this age was lower than that of girls. Therefore, parents may exert more rigorous control on boys, leading to more negative affect.

More notably, this research found that academic achievements predicted positive and negative affect after controlling for demographic variables, indicating that higher academic achievement was associated with more positive affect and less negative affect in China. The combination of more positive affect and less negative affect suggests that higher individual subjective well-being (Bradburn, 1969). Combined with Chang et al. (2003), who investigated life satisfaction in Hong Kong, these results demonstrate that academic achievement influences the affect and cognitive levels of subjective well-being in elementary school children set in Chinese culture. Suldo and Huebner (2006) showed that culture influenced the relationship between academic achievement and subjective well-being. The relationship between subjective well-being and academic achievement is present in cultures that emphasize academic achievement. Under the influence of traditional Confucianism in China, academic achievement is particularly important. Several studies have also confirmed that Asian culture contains some unique values, including emphasizing the importance of hard work and respect for education. These unique values emphasize the academic achievement of Asian students (Sue and Okazaki, 1990). Therefore, in an Asian cultural background, regardless of cognitive or emotional levels, subjective well-being is (unsurprisingly) closely associated with academic achievement.

Self-determination theory suggests that the relationship between academic achievement and the subjective well-being of children may be caused by a variety of complex factors. Self-determination theory proposes three basic psychological demands: autonomy, competence, and relatedness. Fulfillment of these demands is important for both the psychological growth and subjective well-being of individuals (Deci and Ryan, 2000; Ryan and Deci, 2000). Schoolwork is the most important activity of school-age children in China, which to some extent determines the satisfaction of competence and relatedness. Previous studies have shown that the academic achievement of children is closely associated with the sense of both academic and social competence (Anderman and Midgley, 1997). Next, academic achievement can also affect the quality of peer relationships (Wentzel and Caldwell, 1997) as well as teacher-student relationships and parent–child relationships (Zhang, 2013). Therefore, self-determination theory indicates that children’s academic achievement will influence the two basic psychological demands of competence and relatedness, thus influencing individuals’ subjective well-being.

### The Moderating Effect of Parent–School Communication on the Relationship between Emotional Well-Being and Academic Achievement

This study showed that parent–school communication moderates the relationship of both positive and negative affect with academic achievement. In the high scoring and average groups, academic achievement was predictive of emotional well-being, whereas in the low scoring group, no association was observed.

In the Chinese culture, there is little contact between parents and schools. Previous studies have shown that 36% of schools in Hong Kong held two or fewer activities for parents; in 1 week, 18% of teachers did not have time to contact parents; 50% of teachers only had 30 min to communicate with parents (Pang and Watkins, 2000). We found that 76.22% parents reported that they communicated with their children’s school less often than “sometimes” (<3). These results demonstrate that the parent–school communication level of Chinese parents is currently low. However, in addition to a parent–teacher conference organized by the school, parents in the highs coring group might actively communicate with teachers, which reflects parents’ attention to school work and the high expectations they have for their children. By contrast, parents in the low scoring group might only passively participate in the small number of parent–teacher conferences organized by the schools.

The effect exerted by parent–school communication is multi-directional. First, parent–school communication directly impacts children because parents communicate the importance of education and schools to children by means of active and frequent parent–school communication (Lareau, 2000; Pomerantz et al., 2007). When children internalize this parental belief, it may translate to increased attention to academic achievement on the part of the children, thus establishing the relationship between academic achievement and emotional well-being. When parents seldom or only passively communicate with their children’s’ school, children may internalize the passive attitude of parents toward schoolwork, thus diminishing the relationship between academic achievement and emotional well-being.

Next, parents can be influenced by parent–school communication. Parents can obtain academic information regarding their children in schools through parent–school communication, which might lead to a clearer understanding of their children’s academic ability, leading them to adjust their parenting behavior. Chinese parents commonly regard the performance of children as their own report card and largely base their own worth on their children’s achievement (Ng et al., 2014). Therefore, being aware of their child’s academic performance through active parent–school communication will directly impact parents. When their children have better academic results, parents commonly experience higher self-worth and more positive emotions, which might indirectly promote the emotional wellbeing of their children. Children with poor learning abilities typically have low school belonging and poor in-school performance (Svetaz et al., 2000; MacDonald and Leary, 2005). Parents will be extra strict with their children if they hear that their children are not performing well at school. However, when parent–school communication is low, the information obtained is limited. Therefore, the influences of parent–school communication on parents will be weak and not effectively influence children.

Furthermore, parent–school communication can also help teachers to clearly understand parents’ educational goals (Powell et al., 2010). When parents frequently communicate with teachers, parents may pay more attention to their children; therefore, children paying more attention to academic achievements.

### Limitations

As with most previous studies, this study is cross-sectional and cannot indicate a causal relationship between academic achievement and children’s well-being. Therefore, longitudinal studies should examine the moderating role of parents in the relationship between academic achievement and the emotional well-being of children.

Previous studies have shown identity differences in the roles of fathers and mothers (Rogers et al., 2009; Kim and Fong, 2014; Hill, 2015). Thus, father and mother may play different roles in the relationship between academic achievement and children’s emotional well-being. However, fathers answered fewer questionnaires than mothers; thus, investigating the differences between fathers and mothers may be not reliable enough, and further research is required.

## Conclusion and Future Directions

Our results show that the academic achievement of Chinese children predicted their positive and negative affect. In addition, this relationship was moderated by parent–school communication. These results suggest that parents influence the attitude of children toward academic achievement. However, parenting behavior is a double-edged sword in Chinese culture. Active involvement in parent–school communication by parents is conducive to strengthening the attention of children on schoolwork and may enhance academic achievement. However, poor academic achievement in children may induce pessimism and even depression due to the association between well-being and academic achievement. Therefore, future research should investigate the different types of parent–school communication. For example, previous studies have shown that school-initiated and family initiated parent–school communications have opposite effects on children’s academic development (Fan and Williams, 2010). Different forms of parent–school communication may differentially moderate the relationship between academic achievement and the positive and negative affect of children. Use of appropriate parent–school communication by parents to strengthen the relationship between academic achievement and the positive affect of children and inhibit the negative affect caused by poor academic achievement is important to the healthy development of children.

## Author Contributions

BL: found this idea, design of the work, data analyze, paper writing, revising it critically, final approval of the version to be published, Agreement to be accountable for all aspects of the work in ensuring that questions related to the accuracy. HZ: design of the work, data analyze; revising it critically, final approval of the version to be published, integrity of any part of the work are appropriately investigated and resolved. XG: design of the work, paper writing; revising it critically, final approval of the version to be published, integrity of any part of the work are appropriately investigated and resolved. CL: design of the work, paper writing, revising it critically, final approval of the version to be published, integrity of any part of the work are appropriately investigated and resolved. ZL: Overall guidance and took part in all the procedures. LL: Overall guidance and took part in all the procedures.

## Conflict of Interest Statement

The authors declare that the research was conducted in the absence of any commercial or financial relationships that could be construed as a potential conflict of interest.
